# The Value of Percussion

**Published:** 1915-12-11

**Authors:** 


### THE VALUE OF PERCUSSION.

The training necessary to acquire even a moderate degree of competence in the various methods of physical examination involves, as every student knows, a tedious and laborious discipline. Even after the senses have been cultivated to identify the normal and abnormal signs, there remain the difficulties of interpretation, and these, experience alone, will successfully overcome. Of the several methods, probably teachers and pupils will alike admit that percussion presents the most severe demands and the greatest opportunities for error. The obvious lesson is that it should be practised with special care and perseverance. This might be said at any time, but it is worthy of special emphasis in days when, as at present, bedside investigation is in some danger of at least partial eclipse by the more clean-cut decisions of the clinical laboratory. No one, therefore, who is practically concerned with medical education would say anything which would weaken the student's conviction of the unfailing necessity of skill and thoroughness in the methods of physical examination.

Yet it is open to question whether some of the more enthusiastic advocates of percussion do not state the claims of their favourite art in unduly exalted terms. To question their individual and personal accomplishments would be quite unwarranted, for no one can set limits to the degree of skill and delicacy which may be gained in any art by means of sedulous cultivation and practice. It used to be told of a certain epicure that so refined was his sense of taste that in enjoying the leg of a fowl he could distinguish the limb on which the bird had been in the habit of roosting from the less strenuous member; and the principle may quite well apply to percussion. But these fine shades are not for the general capacity. If they can be cultivated by the physician with exceptional gifts and exceptional opportunities well and good; but do not let them be held up as a standard possible to the many. When such a standard is proposed, two unfortunate results follow. One is discouragement to the conscientious student, and the other, following on the first, is an unjust disparagement of the real value of percussion. Admit the special and personal gift and achievement, but do not proclaim them within the reach of the average attainment. Further, it is necessary to say that with all allowances for skill and honest judgment, percussion is a method of examination in which it is easy to deceive oneself, and this more particularly in dealing with the more delicate applications of the art. There is thus a special risk in resting large conclusions on observations made purely by percussion, which is only another way of saying that exaggerations here are particularly dangerous. We read, not without envy, of the achievements of certain authorities, but we are sure that for most of us their claims are beyond the reach of realisation. Perhaps we may find a little consolation in the knowledge that they are not always agreed among themselves.

